# 2,2-Dimethyl-5-(2-nitro­benzyl­idene)-1,3-dioxane-4,6-dione

**DOI:** 10.1107/S1600536812049677

**Published:** 2012-12-08

**Authors:** Fernando García-Álvarez, Nancy Romero, Carlos E. Lobato-García, Joel L. Terán, Angel Mendoza

**Affiliations:** a Centro de Química, Instituto de Ciencias, Benemérita Universidad Autónoma de Puebla, 72570, Puebla, Pue., Mexico; b División Académica de Ciencias Básicas, Universidad Juárez Autónoma de Tabasco, AP 24, 86690, Cuanduacán Tab., Mexico

## Abstract

The asymmetric unit of the title compound, C_13_H_11_NO_6_, contains two mol­ecules in both of which the six-membered 1,3-dioxane-4,6-dione ring shows a screw-boat conformation. The dihedral angles between the best planes through the six-membered rings are 47.8 (2) and 49.8 (2)°. In the crystal, C—H⋯O inter­actions link the mol­ecules, building a supramolecular sheet parallel to the *c* axis.

## Related literature

For general applications of Meldrum’s acid, see: Palasz *et al.* (2007 ▶); Fillion *et al.* (2006 ▶); Mizukami *et al.* (1993 ▶). For the synthesis of heterocyclic compounds, see: Scott & Raston (2000 ▶); Alvim *et al.* (2005 ▶); Fillion & Dumas (2008 ▶). For combinatorial synthesis, see: Shaabani *et al.* (2004 ▶); Wang *et al.* (2007 ▶); Cochard *et al.* (2004 ▶). For puckering parameters, see: Cremer & Pople (1975 ▶). For NMR data, see: Bigi *et al.* (2001 ▶).
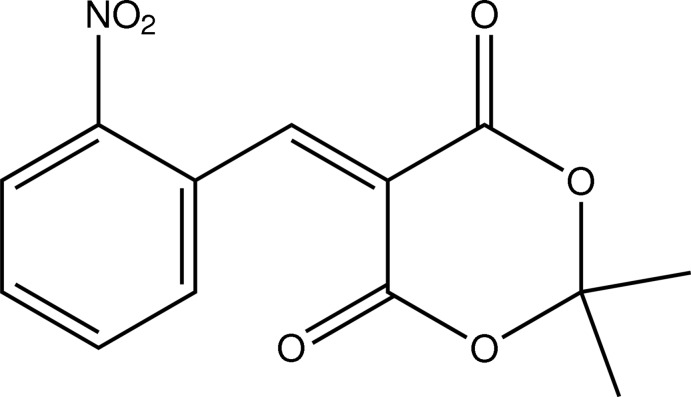


## Experimental

### 

#### Crystal data

C_13_H_11_NO_6_
*M* *_r_* = 277.23
Triclinic, 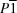

*a* = 10.0240 (4) Å
*b* = 10.4830 (5) Å
*c* = 12.4076 (5) Å
α = 105.385 (4)°
β = 94.669 (3)°
γ = 93.096 (3)°
*V* = 1249.03 (10) Å^3^
*Z* = 4
Mo *K*α radiation
μ = 0.12 mm^−1^
*T* = 130 K
0.39 × 0.22 × 0.14 mm

#### Data collection

Oxford Diffraction Xcalibur (Atlas, Gemini) diffractometer
Absorption correction: analytical (*CrysAlis PRO*; Oxford Diffraction, 2009 ▶) *T*_min_ = 0.968, *T*_max_ = 0.985
8703 measured reflections
4526 independent reflections
3902 reflections with *I* > 2σ(*I*)
*R*_int_ = 0.018

#### Refinement

*R*[*F* ^2^ > 2σ(*F* ^2^)] = 0.035
*wR*(*F* ^2^) = 0.088
*S* = 1.03
4526 reflections
365 parameters
H-atom parameters constrained
Δρ_max_ = 0.22 e Å^−3^
Δρ_min_ = −0.24 e Å^−3^

### 

Data collection: *CrysAlis PRO* (Oxford Diffraction, 2009 ▶); cell refinement: *CrysAlis PRO*; data reduction: *CrysAlis PRO*; program(s) used to solve structure: *SHELXS97* (Sheldrick, 2008 ▶); program(s) used to refine structure: *SHELXL97* (Sheldrick, 2008 ▶); molecular graphics: *ORTEP-3 for Windows* (Farrugia, 2012 ▶); software used to prepare material for publication: *WinGX* (Farrugia, 2012 ▶).

## Supplementary Material

Crystal structure: contains datablock(s) global, I. DOI: 10.1107/S1600536812049677/bt6875sup1.cif

Structure factors: contains datablock(s) I. DOI: 10.1107/S1600536812049677/bt6875Isup2.hkl

Supplementary material file. DOI: 10.1107/S1600536812049677/bt6875Isup3.cml

Additional supplementary materials:  crystallographic information; 3D view; checkCIF report

## Figures and Tables

**Table 1:** Hydrogen-bond geometry (Å, °)

*D*—H⋯*A*	*D*—H	H⋯*A*	*D*⋯*A*	*D*—H⋯*A*
C3—H7⋯O5	0.93	2.43	2.7970 (16)	104
C11—H10⋯O8	0.93	2.45	3.239 (2)	143
C21—H13⋯O5^i^	0.96	2.58	3.4188 (17)	145
C16—H18⋯O12	0.93	2.54	2.8582 (18)	100
C16—H18⋯O2^ii^	0.93	2.55	3.4486 (18)	163
C24—H21⋯O5^iii^	0.93	2.48	3.3676 (19)	160

Symmetry codes: (i) 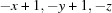; (ii) 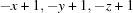; (iii) 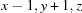.

**Fig. 1.**
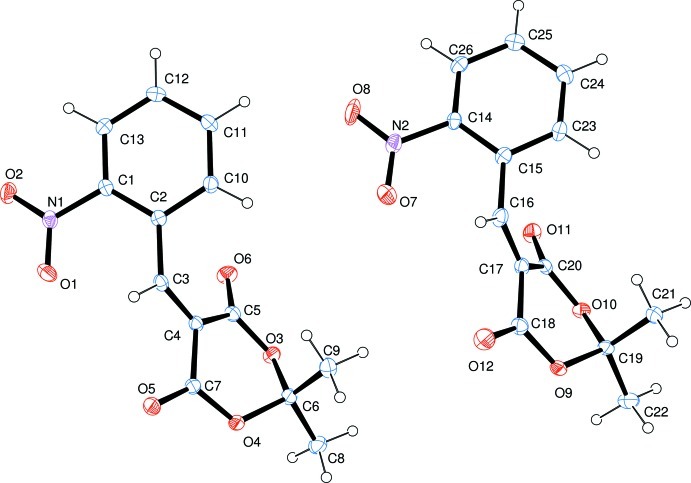
The molecular structure of title compound, with atom labels and 30% probability displacement ellipsoids for non-H atoms.

## supplementary crystallographic information

### Comment

The 5-arylidenes Meldrum's acid derivatives, like title compound, are versatile intermediates in organic synthesis, for example, this type of compounds were used in hetero-Diels-Alder reaction (Palasz *et al.* 2007; Fillion *et al.* 2006 and Mizukami *et al.* 1993), as well as in the synthesis of heterocycle compound, like coumarins (Scott *et al.* 2000 and Alvim *et al.* 2005); γ-Butyrolactones and pyrrole derivatives (Fillion *et al.* 2008). Also, the 5-arylidene derivatives of Meldrum's acid possess an α, β-unsaturated carbonyl system, which is considered as a key building block in combinatorial reaction for three components synthesis (Shaabani *et al.* 2004 and Wang *et al.* 2007), alike four components reaction (Cochard *et al.* 2004). The title compound was obtained by a green Knoevenagel condensation of Meldrum acid with 2-nitrobenzaldehyde employing ultrasonic radiation and water as solvent.

In the title compound C_13_ H_11_ N O_6_, the ASU contains two molecules showing a 2,2-dimethyl-1,3-dioxane-4,6-dione group opposite to *o*-nitrophenyl ring for each molecule. The 1,3-dioxane ring shows a screw-boat conformation with puckering parameters (Cremer & Pople, 1975) *Q* = 0.4807 (14) Å, θ_2_ = 73.12° (17), φ_2_ = 300.32° (17), q_2_ = 0.4600 (14) Å and q_3_ = 0.1396 (14) Å for the six member ring O3/C5/C4/C7/O4/C6 and *Q* = 0.5031 (14) Å, θ_2_ = 103.03 (16)°, φ_2_ = 121.91 (16)°, q_2_ = 0.4901 (14) Å and q_3_ = -0.1135 (14) Å for the six member ring O9/C18/C17/C20/O10/C19. The C=O groups and carbon atom between them show a maximun deviation from mean plane of 0.256 Å for molecule 1 and 0.311 Å for molecule 2. The dihedral angle for *p*-nitrophenyl rings and C=C bond are 56.1 (2)° for molecule 1 (C2/C3/C4/C10) and 60.5 (2)° for molecule 2 (C15/C16/C17/C23). The crystal packing present six intermolecular interactions of the type C—H···O hydrogen bonds (table 1).

### Experimental

In a balloon flask was placed 0.33 mmol of 2-nitrobenzaldehyde, 1 eq. of Meldrum's acid and 3 ml of water. The mixture was subjected to ultrasonic radiation for 37 min. The reaction product was isolated from the aqueous medium by liquid-liquid extraction 3 *x* 10 ml. CH2Cl2 and the crude product recrystallized from CH_2_Cl_2_/Et_2_O (1:1) to give (I) in 72% yield. m.p 117°C. For ultrasonic radiation we employed an ultrasonic bath Cole-Parmer 08890–21. All chemicals were purchased from Sigma-Aldrich. Spectroscopic analysis: ν max/cm-1 (neat KBr) 2925, 1737, 1640, 1600, 1525, 1350, 1380, 1290, 1200, and 725. ^1^H NMR (400 MHz, CDCl_3_): δ (p.p.m.) = 8.80 (s, 1H); 8.3 (dd, 1H); 7.77 (t, 1H); 7.6(t, 1H); 7.5(d, 1H); 1.8 (s, 6H). ^13^C NMR data are in good agreement with those described in the literature (Bigi *et al.*, 2001)

### Refinement

H atoms were placed in geometrical idealized positions and refined as riding on their parent atoms, with C—H = 0.93–0.96 Å and with *U*_iso_(H) = 1.2 *U*_eq_(C) or 1.5 *U*_eq_(methyl C).

### Crystal data

**Table:**

C_13_H_11_NO_6_	*Z* = 4
*M**_r_* = 277.23	*F*(000) = 576
Triclinic, *P*1	*D*_x_ = 1.474 Mg m^−^^3^
*a* = 10.0240 (4) Å	Mo *K*α radiation, λ = 0.71073 Å
*b* = 10.4830 (5) Å	Cell parameters from 5470 reflections
*c* = 12.4076 (5) Å	θ = 3.4–25.2°
α = 105.385 (4)°	µ = 0.12 mm^−^^1^
β = 94.669 (3)°	*T* = 130 K
γ = 93.096 (3)°	Prism, colorless
*V* = 1249.03 (10) Å^3^	0.39 × 0.22 × 0.14 mm

### Data collection

**Table:**

Oxford Diffraction Xcalibur (Atlas, Gemini) diffractometer	4526 independent reflections
Graphite monochromator	3902 reflections with *I* > 2σ(*I*)
Detector resolution: 10.4685 pixels mm^-1^	*R*_int_ = 0.018
ω scans	θ_max_ = 25.3°, θ_min_ = 3.4°
Absorption correction: analytical (*CrysAlis PRO*; Oxford Diffraction, 2009)	*h* = −12→12
*T*_min_ = 0.968, *T*_max_ = 0.985	*k* = −10→12
8703 measured reflections	*l* = −13→14

### Refinement

**Table:**

Refinement on *F*^2^	Primary atom site location: structure-invariant direct methods
Least-squares matrix: full	Secondary atom site location: difference Fourier map
*R*[*F*^2^ > 2σ(*F*^2^)] = 0.035	Hydrogen site location: inferred from neighbouring sites
*wR*(*F*^2^) = 0.088	H-atom parameters constrained
*S* = 1.03	*w* = 1/[σ^2^(*F*_o_^2^) + (0.0397*P*)^2^ + 0.3347*P*] where *P* = (*F*_o_^2^ + 2*F*_c_^2^)/3
4526 reflections	(Δ/σ)_max_ < 0.001
365 parameters	Δρ_max_ = 0.22 e Å^−^^3^
0 restraints	Δρ_min_ = −0.24 e Å^−^^3^

### Special details

**Table:**

Geometry. All s.u.'s (except the s.u. in the dihedral angle between two l.s. planes) are estimated using the full covariance matrix. The cell s.u.'s are taken into account individually in the estimation of s.u.'s in distances, angles and torsion angles; correlations between s.u.'s in cell parameters are only used when they are defined by crystal symmetry. An approximate (isotropic) treatment of cell s.u.'s is used for estimating s.u.'s involving l.s. planes.
Refinement. Refinement of *F*^2^ against ALL reflections. The weighted *R*-factor *wR* and goodness of fit *S* are based on *F*^2^, conventional *R*-factors *R* are based on *F*, with *F* set to zero for negative *F*^2^. The threshold expression of *F*^2^ > 2σ(*F*^2^) is used only for calculating *R*-factors(gt) *etc*. and is not relevant to the choice of reflections for refinement. *R*-factors based on *F*^2^ are statistically about twice as large as those based on *F*, and *R*- factors based on ALL data will be even larger.

### Fractional atomic coordinates and isotropic or equivalent isotropic displacement parameters (Å^2^)

**Table:**

	*x*	*y*	*z*	*U*_iso_*/*U*_eq_	
O1	0.61144 (10)	0.10481 (11)	0.54216 (9)	0.0347 (3)	
O2	0.53572 (11)	0.09437 (10)	0.69780 (9)	0.0351 (3)	
O6	0.23911 (10)	0.16149 (11)	0.32752 (8)	0.0334 (3)	
O5	0.68803 (9)	0.16562 (11)	0.25320 (8)	0.0293 (2)	
O3	0.29864 (9)	0.16222 (10)	0.16040 (8)	0.0260 (2)	
O4	0.52511 (9)	0.16412 (10)	0.12250 (8)	0.0255 (2)	
N1	0.54108 (11)	0.14413 (11)	0.61912 (10)	0.0241 (3)	
C2	0.44468 (13)	0.30112 (13)	0.52086 (11)	0.0203 (3)	
C1	0.46117 (13)	0.25651 (13)	0.61755 (11)	0.0203 (3)	
C13	0.40048 (14)	0.31165 (14)	0.71352 (11)	0.0240 (3)	
H8	0.4137	0.2793	0.7762	0.029*	
C12	0.32022 (14)	0.41500 (14)	0.71558 (12)	0.0264 (3)	
H9	0.2803	0.4543	0.7801	0.032*	
C11	0.29991 (14)	0.45936 (14)	0.62034 (12)	0.0263 (3)	
H10	0.2446	0.5279	0.6207	0.032*	
C10	0.36058 (13)	0.40341 (14)	0.52468 (12)	0.0243 (3)	
H11	0.345	0.4347	0.4616	0.029*	
C3	0.51994 (13)	0.25533 (13)	0.42226 (11)	0.0215 (3)	
H7	0.6126	0.2577	0.4371	0.026*	
C4	0.47179 (13)	0.21081 (13)	0.31422 (11)	0.0209 (3)	
C5	0.32781 (14)	0.17904 (14)	0.27213 (12)	0.0241 (3)	
C7	0.57212 (13)	0.18052 (14)	0.23029 (11)	0.0222 (3)	
C6	0.39619 (13)	0.21135 (15)	0.09837 (12)	0.0243 (3)	
C8	0.35526 (15)	0.14531 (17)	−0.02350 (12)	0.0330 (4)	
H1	0.2719	0.1764	−0.0458	0.05*	
H2	0.3444	0.0509	−0.0351	0.05*	
H3	0.4233	0.1663	−0.0676	0.05*	
C9	0.40534 (15)	0.36051 (15)	0.12822 (13)	0.0302 (3)	
H4	0.3197	0.3899	0.1091	0.045*	
H5	0.4716	0.3912	0.0872	0.045*	
H6	0.4306	0.3957	0.2074	0.045*	
O7	0.21049 (10)	0.63351 (11)	0.34639 (9)	0.0367 (3)	
O8	0.12315 (15)	0.64061 (17)	0.49838 (11)	0.0703 (5)	
O11	−0.11562 (10)	0.62735 (10)	0.09585 (9)	0.0305 (2)	
O12	0.33877 (10)	0.68213 (12)	0.06066 (9)	0.0380 (3)	
O10	−0.04438 (9)	0.64388 (9)	−0.06351 (8)	0.0235 (2)	
O9	0.18763 (9)	0.66645 (9)	−0.08312 (8)	0.0230 (2)	
N2	0.12876 (12)	0.67424 (13)	0.41284 (10)	0.0297 (3)	
C15	0.04065 (13)	0.81061 (14)	0.29236 (11)	0.0236 (3)	
C23	−0.05066 (14)	0.90041 (15)	0.27642 (12)	0.0282 (3)	
H22	−0.0497	0.9318	0.2131	0.034*	
C24	−0.14323 (14)	0.94452 (15)	0.35226 (13)	0.0291 (3)	
H21	−0.2031	1.0049	0.3395	0.035*	
C25	−0.14677 (14)	0.89901 (14)	0.44700 (12)	0.0267 (3)	
H20	−0.2091	0.9285	0.4978	0.032*	
C26	−0.05768 (14)	0.80991 (14)	0.46575 (11)	0.0246 (3)	
H19	−0.0596	0.7782	0.5289	0.029*	
C14	0.03491 (13)	0.76803 (13)	0.38935 (11)	0.0218 (3)	
C16	0.14020 (14)	0.77088 (15)	0.21051 (12)	0.0263 (3)	
H18	0.2304	0.7894	0.2375	0.032*	
C17	0.11190 (13)	0.71080 (14)	0.10134 (12)	0.0232 (3)	
C18	0.22400 (14)	0.68726 (14)	0.02790 (12)	0.0256 (3)	
C20	−0.02527 (13)	0.65966 (13)	0.04834 (11)	0.0220 (3)	
C19	0.05674 (13)	0.70168 (14)	−0.11726 (11)	0.0223 (3)	
C21	0.05316 (14)	0.85027 (14)	−0.08805 (12)	0.0265 (3)	
H12	−0.0339	0.8722	−0.1125	0.04*	
H13	0.1202	0.8868	−0.1249	0.04*	
H14	0.0709	0.8865	−0.0082	0.04*	
C22	0.02837 (15)	0.63545 (16)	−0.24028 (12)	0.0321 (4)	
H15	−0.0618	0.6486	−0.2651	0.048*	
H16	0.0385	0.5421	−0.2539	0.048*	
H17	0.0902	0.6731	−0.2808	0.048*	

### Atomic displacement parameters (Å^2^)

**Table:**

	*U*^11^	*U*^22^	*U*^33^	*U*^12^	*U*^13^	*U*^23^
O1	0.0352 (6)	0.0324 (6)	0.0391 (6)	0.0151 (5)	0.0113 (5)	0.0096 (5)
O2	0.0461 (7)	0.0304 (6)	0.0325 (6)	0.0077 (5)	−0.0012 (5)	0.0158 (5)
O6	0.0196 (5)	0.0511 (7)	0.0312 (6)	0.0000 (5)	0.0079 (4)	0.0131 (5)
O5	0.0189 (5)	0.0423 (6)	0.0304 (6)	0.0084 (5)	0.0056 (4)	0.0143 (5)
O3	0.0199 (5)	0.0345 (6)	0.0234 (5)	−0.0002 (4)	0.0016 (4)	0.0084 (4)
O4	0.0213 (5)	0.0358 (6)	0.0218 (5)	0.0085 (4)	0.0049 (4)	0.0097 (4)
N1	0.0233 (6)	0.0215 (6)	0.0259 (7)	0.0011 (5)	−0.0038 (5)	0.0056 (5)
C2	0.0161 (6)	0.0212 (7)	0.0232 (7)	−0.0007 (5)	0.0007 (5)	0.0059 (6)
C1	0.0171 (7)	0.0173 (7)	0.0252 (7)	−0.0004 (5)	−0.0016 (5)	0.0050 (5)
C13	0.0264 (7)	0.0248 (7)	0.0208 (7)	−0.0013 (6)	0.0008 (6)	0.0072 (6)
C12	0.0267 (8)	0.0246 (7)	0.0262 (8)	0.0015 (6)	0.0084 (6)	0.0026 (6)
C11	0.0238 (7)	0.0232 (7)	0.0332 (8)	0.0067 (6)	0.0070 (6)	0.0075 (6)
C10	0.0226 (7)	0.0270 (8)	0.0264 (8)	0.0038 (6)	0.0034 (6)	0.0121 (6)
C3	0.0171 (7)	0.0225 (7)	0.0274 (8)	0.0032 (6)	0.0035 (5)	0.0105 (6)
C4	0.0188 (7)	0.0222 (7)	0.0244 (7)	0.0039 (6)	0.0047 (5)	0.0098 (6)
C5	0.0212 (7)	0.0260 (8)	0.0259 (8)	0.0044 (6)	0.0037 (6)	0.0076 (6)
C7	0.0219 (7)	0.0232 (7)	0.0231 (7)	0.0028 (6)	0.0042 (6)	0.0084 (6)
C6	0.0164 (7)	0.0332 (8)	0.0259 (8)	0.0042 (6)	0.0038 (5)	0.0116 (6)
C8	0.0300 (8)	0.0439 (10)	0.0249 (8)	0.0053 (7)	0.0005 (6)	0.0091 (7)
C9	0.0273 (8)	0.0311 (8)	0.0343 (9)	0.0036 (7)	0.0014 (6)	0.0126 (7)
O7	0.0308 (6)	0.0402 (7)	0.0411 (7)	0.0121 (5)	0.0056 (5)	0.0124 (5)
O8	0.0808 (10)	0.1092 (13)	0.0500 (9)	0.0550 (10)	0.0230 (7)	0.0579 (9)
O11	0.0240 (5)	0.0348 (6)	0.0374 (6)	−0.0012 (5)	0.0082 (4)	0.0173 (5)
O12	0.0183 (5)	0.0609 (8)	0.0386 (6)	0.0095 (5)	0.0038 (4)	0.0185 (6)
O10	0.0185 (5)	0.0263 (5)	0.0243 (5)	−0.0002 (4)	0.0039 (4)	0.0045 (4)
O9	0.0186 (5)	0.0264 (5)	0.0246 (5)	0.0066 (4)	0.0055 (4)	0.0061 (4)
N2	0.0281 (7)	0.0360 (7)	0.0262 (7)	0.0032 (6)	−0.0032 (5)	0.0120 (6)
C15	0.0199 (7)	0.0275 (8)	0.0230 (7)	−0.0015 (6)	−0.0011 (5)	0.0077 (6)
C23	0.0289 (8)	0.0315 (8)	0.0282 (8)	0.0021 (6)	0.0000 (6)	0.0158 (6)
C24	0.0250 (8)	0.0255 (8)	0.0363 (9)	0.0039 (6)	−0.0005 (6)	0.0080 (6)
C25	0.0214 (7)	0.0265 (8)	0.0289 (8)	−0.0022 (6)	0.0031 (6)	0.0023 (6)
C26	0.0245 (7)	0.0282 (8)	0.0202 (7)	−0.0054 (6)	−0.0004 (5)	0.0074 (6)
C14	0.0192 (7)	0.0234 (7)	0.0220 (7)	−0.0007 (6)	−0.0044 (5)	0.0069 (6)
C16	0.0203 (7)	0.0346 (8)	0.0275 (8)	0.0028 (6)	0.0009 (6)	0.0149 (6)
C17	0.0190 (7)	0.0266 (7)	0.0279 (8)	0.0044 (6)	0.0049 (6)	0.0131 (6)
C18	0.0215 (8)	0.0282 (8)	0.0293 (8)	0.0037 (6)	0.0048 (6)	0.0103 (6)
C20	0.0217 (7)	0.0189 (7)	0.0276 (8)	0.0058 (6)	0.0051 (6)	0.0084 (6)
C19	0.0163 (7)	0.0279 (8)	0.0239 (7)	0.0040 (6)	0.0054 (5)	0.0078 (6)
C21	0.0263 (8)	0.0278 (8)	0.0285 (8)	0.0071 (6)	0.0073 (6)	0.0106 (6)
C22	0.0259 (8)	0.0423 (9)	0.0253 (8)	0.0048 (7)	0.0044 (6)	0.0031 (7)

### Geometric parameters (Å, º)

**Table:**

O1—N1	1.2293 (15)	O7—N2	1.2223 (15)
O2—N1	1.2263 (15)	O8—N2	1.2083 (16)
O6—C5	1.2024 (16)	O11—C20	1.2009 (16)
O5—C7	1.2018 (16)	O12—C18	1.1974 (17)
O3—C5	1.3554 (17)	O10—C20	1.3497 (16)
O3—C6	1.4464 (16)	O10—C19	1.4482 (16)
O4—C7	1.3442 (16)	O9—C18	1.3521 (17)
O4—C6	1.4455 (16)	O9—C19	1.4430 (16)
N1—C1	1.4634 (17)	N2—C14	1.4642 (18)
C2—C10	1.3928 (19)	C15—C23	1.388 (2)
C2—C1	1.3996 (19)	C15—C14	1.3944 (19)
C2—C3	1.4745 (18)	C15—C16	1.4785 (19)
C1—C13	1.3830 (19)	C23—C24	1.385 (2)
C13—C12	1.380 (2)	C23—H22	0.93
C13—H8	0.93	C24—C25	1.383 (2)
C12—C11	1.385 (2)	C24—H21	0.93
C12—H9	0.93	C25—C26	1.377 (2)
C11—C10	1.3818 (19)	C25—H20	0.93
C11—H10	0.93	C26—C14	1.3853 (19)
C10—H11	0.93	C26—H19	0.93
C3—C4	1.3364 (19)	C16—C17	1.333 (2)
C3—H7	0.93	C16—H18	0.93
C4—C5	1.4831 (19)	C17—C20	1.4820 (19)
C4—C7	1.4921 (18)	C17—C18	1.4938 (19)
C6—C8	1.499 (2)	C19—C22	1.4961 (19)
C6—C9	1.504 (2)	C19—C21	1.506 (2)
C8—H1	0.96	C21—H12	0.96
C8—H2	0.96	C21—H13	0.96
C8—H3	0.96	C21—H14	0.96
C9—H4	0.96	C22—H15	0.96
C9—H5	0.96	C22—H16	0.96
C9—H6	0.96	C22—H17	0.96
			
C5—O3—C6	119.09 (10)	C20—O10—C19	118.89 (10)
C7—O4—C6	118.75 (10)	C18—O9—C19	118.26 (10)
O2—N1—O1	123.04 (12)	O8—N2—O7	122.23 (13)
O2—N1—C1	118.41 (11)	O8—N2—C14	118.42 (12)
O1—N1—C1	118.54 (11)	O7—N2—C14	119.35 (12)
C10—C2—C1	116.46 (12)	C23—C15—C14	116.39 (13)
C10—C2—C3	119.36 (12)	C23—C15—C16	119.29 (12)
C1—C2—C3	123.91 (12)	C14—C15—C16	124.26 (13)
C13—C1—C2	122.65 (12)	C24—C23—C15	121.82 (13)
C13—C1—N1	116.92 (12)	C24—C23—H22	119.1
C2—C1—N1	120.40 (12)	C15—C23—H22	119.1
C12—C13—C1	119.50 (13)	C25—C24—C23	120.14 (14)
C12—C13—H8	120.3	C25—C24—H21	119.9
C1—C13—H8	120.3	C23—C24—H21	119.9
C13—C12—C11	119.10 (13)	C26—C25—C24	119.73 (13)
C13—C12—H9	120.5	C26—C25—H20	120.1
C11—C12—H9	120.5	C24—C25—H20	120.1
C10—C11—C12	121.01 (13)	C25—C26—C14	119.18 (13)
C10—C11—H10	119.5	C25—C26—H19	120.4
C12—C11—H10	119.5	C14—C26—H19	120.4
C11—C10—C2	121.25 (13)	C26—C14—C15	122.73 (13)
C11—C10—H11	119.4	C26—C14—N2	117.53 (12)
C2—C10—H11	119.4	C15—C14—N2	119.74 (12)
C4—C3—C2	128.19 (12)	C17—C16—C15	125.68 (13)
C4—C3—H7	115.9	C17—C16—H18	117.2
C2—C3—H7	115.9	C15—C16—H18	117.2
C3—C4—C5	125.32 (12)	C16—C17—C20	123.63 (12)
C3—C4—C7	116.92 (12)	C16—C17—C18	119.06 (12)
C5—C4—C7	117.57 (12)	C20—C17—C18	117.25 (12)
O6—C5—O3	119.04 (12)	O12—C18—O9	120.03 (12)
O6—C5—C4	125.42 (13)	O12—C18—C17	124.61 (13)
O3—C5—C4	115.44 (11)	O9—C18—C17	115.32 (11)
O5—C7—O4	119.64 (12)	O11—C20—O10	119.26 (12)
O5—C7—C4	124.14 (12)	O11—C20—C17	125.36 (13)
O4—C7—C4	116.15 (11)	O10—C20—C17	115.31 (11)
O4—C6—O3	109.08 (10)	O9—C19—O10	109.68 (10)
O4—C6—C8	105.46 (11)	O9—C19—C22	106.65 (11)
O3—C6—C8	106.45 (11)	O10—C19—C22	105.78 (11)
O4—C6—C9	110.69 (11)	O9—C19—C21	110.39 (11)
O3—C6—C9	110.72 (11)	O10—C19—C21	110.60 (11)
C8—C6—C9	114.17 (12)	C22—C19—C21	113.56 (12)
C6—C8—H1	109.5	C19—C21—H12	109.5
C6—C8—H2	109.5	C19—C21—H13	109.5
H1—C8—H2	109.5	H12—C21—H13	109.5
C6—C8—H3	109.5	C19—C21—H14	109.5
H1—C8—H3	109.5	H12—C21—H14	109.5
H2—C8—H3	109.5	H13—C21—H14	109.5
C6—C9—H4	109.5	C19—C22—H15	109.5
C6—C9—H5	109.5	C19—C22—H16	109.5
H4—C9—H5	109.5	H15—C22—H16	109.5
C6—C9—H6	109.5	C19—C22—H17	109.5
H4—C9—H6	109.5	H15—C22—H17	109.5
H5—C9—H6	109.5	H16—C22—H17	109.5
			
C10—C2—C1—C13	−1.4 (2)	C14—C15—C23—C24	−0.5 (2)
C3—C2—C1—C13	172.63 (13)	C16—C15—C23—C24	−177.84 (13)
C10—C2—C1—N1	176.31 (11)	C15—C23—C24—C25	−0.1 (2)
C3—C2—C1—N1	−9.7 (2)	C23—C24—C25—C26	0.2 (2)
O2—N1—C1—C13	9.41 (18)	C24—C25—C26—C14	0.4 (2)
O1—N1—C1—C13	−169.89 (12)	C25—C26—C14—C15	−1.1 (2)
O2—N1—C1—C2	−168.41 (12)	C25—C26—C14—N2	179.42 (12)
O1—N1—C1—C2	12.29 (18)	C23—C15—C14—C26	1.2 (2)
C2—C1—C13—C12	−0.1 (2)	C16—C15—C14—C26	178.33 (13)
N1—C1—C13—C12	−177.85 (12)	C23—C15—C14—N2	−179.42 (12)
C1—C13—C12—C11	1.4 (2)	C16—C15—C14—N2	−2.2 (2)
C13—C12—C11—C10	−1.2 (2)	O8—N2—C14—C26	−1.8 (2)
C12—C11—C10—C2	−0.3 (2)	O7—N2—C14—C26	178.86 (13)
C1—C2—C10—C11	1.6 (2)	O8—N2—C14—C15	178.79 (15)
C3—C2—C10—C11	−172.72 (13)	O7—N2—C14—C15	−0.60 (19)
C10—C2—C3—C4	−56.1 (2)	C23—C15—C16—C17	−60.5 (2)
C1—C2—C3—C4	130.06 (16)	C14—C15—C16—C17	122.41 (16)
C2—C3—C4—C5	−9.8 (2)	C15—C16—C17—C20	−7.9 (2)
C2—C3—C4—C7	175.41 (13)	C15—C16—C17—C18	174.98 (13)
C6—O3—C5—O6	166.16 (13)	C19—O9—C18—O12	−166.58 (13)
C6—O3—C5—C4	−17.28 (17)	C19—O9—C18—C17	15.59 (17)
C3—C4—C5—O6	−16.9 (2)	C16—C17—C18—O12	22.6 (2)
C7—C4—C5—O6	157.82 (14)	C20—C17—C18—O12	−154.70 (14)
C3—C4—C5—O3	166.76 (13)	C16—C17—C18—O9	−159.71 (13)
C7—C4—C5—O3	−18.49 (18)	C20—C17—C18—O9	23.02 (18)
C6—O4—C7—O5	−165.93 (13)	C19—O10—C20—O11	170.43 (12)
C6—O4—C7—C4	16.79 (17)	C19—O10—C20—C17	−12.53 (17)
C3—C4—C7—O5	16.9 (2)	C16—C17—C20—O11	−24.8 (2)
C5—C4—C7—O5	−158.32 (14)	C18—C17—C20—O11	152.33 (14)
C3—C4—C7—O4	−165.97 (12)	C16—C17—C20—O10	158.34 (13)
C5—C4—C7—O4	18.83 (18)	C18—C17—C20—O10	−24.51 (17)
C7—O4—C6—O3	−50.14 (15)	C18—O9—C19—O10	−50.65 (15)
C7—O4—C6—C8	−164.12 (12)	C18—O9—C19—C22	−164.75 (12)
C7—O4—C6—C9	71.93 (15)	C18—O9—C19—C21	71.46 (14)
C5—O3—C6—O4	50.57 (15)	C20—O10—C19—O9	49.17 (15)
C5—O3—C6—C8	163.91 (12)	C20—O10—C19—C22	163.83 (11)
C5—O3—C6—C9	−71.47 (15)	C20—O10—C19—C21	−72.81 (14)

### Hydrogen-bond geometry (Å, º)

**Table:**

*D*—H···*A*	*D*—H	H···*A*	*D*···*A*	*D*—H···*A*
C3—H7···O5	0.93	2.43	2.7970 (16)	104
C11—H10···O8	0.93	2.45	3.239 (2)	143
C21—H13···O5^i^	0.96	2.58	3.4188 (17)	145
C16—H18···O12	0.93	2.54	2.8582 (18)	100
C16—H18···O2^ii^	0.93	2.55	3.4486 (18)	163
C24—H21···O5^iii^	0.93	2.48	3.3676 (19)	160

Symmetry codes: (i) −*x*+1, −*y*+1, −*z*; (ii) −*x*+1, −*y*+1, −*z*+1; (iii) *x*−1, *y*+1, *z*.
